# Young adults’ circulating FGF23 and α-klotho and their relationship with habitual dietary acid load and phosphorus intake during growth

**DOI:** 10.1038/s41598-024-79636-0

**Published:** 2024-11-13

**Authors:** Luciana Peixoto Franco, Seyedeh-Masomeh Derakhshandeh-Rishehri, Ute Nöthlings, Michaela F. Hartmann, Christian Herder, Hermann Kalhoff, Stefan A. Wudy, Thomas Remer

**Affiliations:** 1 DONALD Study Center, Nutritional Epidemiology, Institute of Nutrition and Food Science, University of Bonn, Heinstück 11, 44225 Dortmund, Germany; 2 Institute of Nutrition and Food Sciences, Nutritional Epidemiology, University of Bonn, Bonn, Germany; 3 Laboratory for Translational Hormone Analytics, Peptide Hormone & Immunoassay Unit, Pediatric Endocrinology & Diabetology, Center of Child and Adolescent Medicine, Justus Liebig University, Giessen, Germany; 4 Institute for Clinical Diabetology, German Diabetes Center (DDZ), Leibniz Center for Diabetes Research at Heinrich Heine University Düsseldorf, Düsseldorf, Germany; 5 German Center for Diabetes Research (DZD), Partner Düsseldorf, München-Neuherberg, Germany; 6 Department of Endocrinology and Diabetology, Medical Faculty, University Hospital Düsseldorf, Heinrich Heine University Düsseldorf, Düsseldorf, Germany; 7 Research Department of Child Nutrition, St. Josef-Hospital, University Hospital of Pediatrics and Adolescent Medicine, Ruhr-University Bochum, Bochum, Germany; 8 Pediatric Clinic Dortmund, Dortmund, Germany

**Keywords:** Fibroblast growth factor 23, Alpha-klotho, Phosphate, Net acid excretion, Urinary potential renal acid load, Habitual dietary acid load, Biomarkers, Endocrinology

## Abstract

The bone-derived hormone FGF23, primarily secreted by osteocytes, is a major player in the regulation of phosphate homeostasis. It becomes upregulated by increased circulating phosphate concentration, e.g. due to elevations in phosphorus intake (P-In) or alterations in habitual dietary acid load. The present study aimed to investigate whether long-term endogenous acid production or a habitual high phosphorus intake during childhood and adolescence may be prospectively related with altered adult levels of FGF23 and the FGF23-related metabolite α-klotho. Urinary phosphate excretion (PO4-Ex), net acid excretion (NAE), and potential renal acid load (uPRAL) were analyzed in 24-h urine samples (*n* = 3369) collected from 343 healthy 3–17 years old participants of the DONALD Study (Dortmund, Germany) to assess, biomarker-based, P-In and habitual dietary acid load. Circulating FGF23, α-klotho, and further blood parameters were additionally examined in young adulthood. Individual means of standard-deviation-scores were calculated for 24-h urinary biomarker excretions and anthropometrics longitudinally determined between ages 3–17 years. Multivariable linear regression was used to analyze the prospective relations of pre-adulthood PO4-Ex, NAE, and uPRAL with the adulthood outcomes FGF23 and α-klotho. After adjusting for growth period-related covariates and adulthood confounders only for P-In during growth, i.e., PO4-Ex, but not for NAE and uPRAL, a positive association (*p* = 0.03) with FGF23 was observed. Neither PO4-Ex, nor NAE or uPRAL were associated with soluble α-klotho levels in adulthood. The prospective relationships of long-term assessed 24-h phosphaturia and habitual dietary acid load during growth with adult circulating, phosphate-adjusted FGF23 strongly suggest that children´s habitually higher P-In does unfavorably affect adult FGF23–α-klotho axis.

**Supplementary Information:**

The online version contains supplementary material available at 10.1038/s41598-024-79636-0.

## Introduction

Fibroblast growth factor 23 (FGF23) is a phosphaturic hormone primarily produced by bone tissue^1^, highly expressed in osteocytes and osteoblasts. Along with parathyroid hormone (PTH), FGF23 is essential for regulating phosphate homeostasis^2,3^. With a habitual high phosphorus intake, phosphate blood concentrations also rise, physiologically leading not only to an increase of PTH, but also of FGF23. Both hormones are mandatorily involved in normalizing and reducing elevated serum phosphate levels^2^. These phosphate reductions are achieved through inhibition of proximal tubular phosphate reabsorption in the kidney by FGF23 and PTH^3^. FGF23, unlike PTH, also reduces phosphate absorption in the intestine^2,4^. When blood phosphate levels increase, bone cells raise FGF23 production and thus augment endocrine effects in intestine and kidneys via binding to the FGF receptor that requires the mandatory co-receptor α-klotho for appropriate FGF23-signaling^2,5^. α-klotho is a transmembrane protein, predominantly expressed in renal tubular epithelial cells^6^, of which its truncated cleaved form circulates as a hormone (secreted α-klotho) that impacts on insulin and other signaling pathways^7^. α-klotho has also been identified as a longevity factor that declines with aging^7^. As one of the major regulators of phosphaturia FGF23 (together with α-klotho) acts via reducing the expression of the type II Na^+^-dependent phosphate co-transporters NaPi-2a and NaPi-2c which – in the renal proximal tubules – are responsible for the active reabsorption of phosphate from tubular urine.

An important environment-related factor influencing FGF23 secretion and renal phosphate reabsorption is dietary phosphorus intake. High phosphate intake for more than one day has been shown to significantly raise phosphate blood levels, circulating FGF23, and renal phosphate excretion^8,9^. However, neither acute short-term increases of serum phosphate levels^10^nor moderate variations of phosphorus intake with normal diets^11^appear to markedly alter FGF23 blood concentrations as has been shown in adolescent girls on their usual diets^11^. Up to now no data are available on whether a habitual higher intake of phosphorus during growth may alter FGF23 levels and the phosphate-FGF23 set point in the long-term. Therefore, we aimed to examine – specifically biomarker-based – the prospective relationship of habitual phosphorus intake during childhood and adolescence with circulating concentrations of FGF23 and α-klotho in later adulthood. For this, measurements of daily phosphate excretion were performed in repeatedly collected 24-h urine samples in 3–17 year old participants of the DOrtmund Nutritional and Anthropometric Longitudinally Designed (DONALD) Study. In healthy individuals, not consuming extreme diets, 24-hour urinary phosphate excretion serves as a reliable biomarker for estimating total phosphorus intake^12^. Foods contain phosphorus either organically bound or as inorganic phosphate. The organically bound phosphorus is intestinally cleaved and oxidized before actively or passively absorbed as phosphate^2^ and the later thus represents a suitable nutritional biomarker.

Although metabolic acidosis has been repeatedly reported to be associated with elevated FGF23 levels in CKD patients^13^, administration of high acid loads to healthy people may rather reduce circulating FGF23^14^, since high acidic stimuli independently raise renal phosphaturia^15^thus providing immediate buffer capacity for renal proton elimination^3^. Therefore, our second aim was to additionally examine whether children and adolescents with higher daily acid loads, assessed via 24-h renal net acid excretion (NAE) and urinary potential renal acid load (uPRAL) measurements, may show an altered adult FGF23 level independent of long-term P-In and plasma phosphate.

## Materials and methods

### Study population

In the present prospective longitudinal study, healthy children and adolescents within the age range of 3–17 years from the ongoing Dortmund Nutritional and Anthropometric Longitudinally Designed (DONALD) study were examined.

The DONALD study is an open cohort study which started in 1985 in Dortmund (Germany) and collects information on diet, anthropometrics, metabolism, and development of healthy volunteers from infancy until adulthood^16,17^. After the age of two years, examinations of children are scheduled in yearly intervals and consist of medical evaluations, anthropometric measurements, 24-h urine collections, and 3-day weighed dietary records. Additionally, from 18 years onward, fasting blood samples are also taken in multiannual intervals^16,17^. All assessments were performed with written informed consent of parents and/or legal guardian and grown-up children, and the study protocol was approved by the ethics committee of the University of Bonn, Germany (approval numbers. 098/06 and 185/20). All experiments were performed in accordance with the guidelines of the Declaration of Helsinki.

Participants of the DONALD study for whom a minimum of 5 eligible 24-h urine collections were available during growth, at least 2 within childhood (3–8 y) and at least 3 within adolescence (9–17 y) were considered, resulting in an initial number of 578 participants. Those participants who had at least five 24-hour urines with completed measurements of PO4, and a minimum of three 24-hour urines with completed NAE measurements between age 3 and 17 years were further selected (*n* = 523). Of those 523 participants, all subjects (*n* = 343) were finally included in whom at least one blood collection had been performed within the age range of 18 to 35 years. Blood samples of DONALD participants already older than 35 years were not analyzed for the purpose of examining a rather homogenous “young adulthood sample”. In case that more than one blood sample had been taken until age 35, participants’ most recent blood collection date was used for the measurement of FGF23 and α-klotho. Follow-up duration between children’s and youths’ median time point of 24-h urine collection and the date of blood sampling was 10.2 years.

For an additional sensitivity analysis including also biomarkers of inflammation, a total of 264 individuals with additional available blood measurements of these biomarkers were considered.

## Anthropometric measurements

Anthropometric measurements were conducted by trained nurses with participants dressed in underwear and without shoes, in accordance with standardized procedures^17,18^. Standing height was assessed to the nearest 0.1 cm with a digital wall-mounted stadiometer (Harpenden, Holtain Ltd, Crymych, UK) and body weight was determined to the nearest 0.1 kg with an electronic scale (Seca 753E; Seca Weighing and Measuring Systems, Hamburg, Germany). Height and body weight data were used to calculate body mass index (BMI) and body surface area (BSA), according to the following formulas: BMI (kg/m²) = weight/height^2^ and BSA (m²) = 0.007184 x height (cm)^0.725^ x weight (kg)^0.425^as reported by Du Bois and Du Bois^19^.

Measurements of skinfold thicknesses were performed at both sides, triceps and subscapular, to the nearest 0.1 mm with a Holtain caliper. Subsequently, body fat percentage (BF%) was determined by using the skinfold data and formulas of Slaughter et al^20^.. Fat mass index (FMI) was calculated by dividing fat mass by height^2^ (kg/m^2^).

## Urinary measurements

Each child and their parents received personal and written instructions on how to collect the 24-h urine sample according to standardized procedures. Urine samples were self-collected at home using preservative free, Extran-cleaned (Extran, MA03; Merck) 1-L plastic containers. Samples were stored at -18 to -20 °C until being thawed for analysis^21^.

Urinary creatinine concentrations were quantified with a creatinine analyzer (Beckman-2; Beckman Instruments, Fullerton, CA), based on the kinetic Jaffé procedure. Samples with a daily creatinine excret**i**on < 0.1 mmol/kg were not considered in the analysis^22^in order to minimize possible errors of urine collection. The urease-Berthelot method (Randox Laboratories, Crumlin, UK) was used to determine urinary urea. Uric acid (mmol/L) was analyzed by the uricase method using the Uric Acid plus kit (Roche Diagnostics GmbH, Mannheim, Germany)^21^.

The NAE analytes ammonium (mmol/L), titratable acidity (mEq/L), and bicarbonate (mmol/L) were quantified by the three phase acid–base titration method^23^ using a Mettler Toledo endpoint titrator (Mettler Toledo, Giessen, Germany), which was also applied to quantify the urinary pH of the 24-h urine samples. NAE was then calculated by summing ammonium and titratable acid minus bicarbonate.

The mineral cations sodium, potassium, calcium, and magnesium, i.e. the alkalizing components of the urinary potential renal acid load (uPRAL), were quantified by flame atomic absorption spectrometry (Perkin Elmer 1100 Spectrometer; Perkin Elmer, Überlingen, Germany). Urinary excretion rates of the anions phosphate, sulfate and chloride were quantified using a Dionex 2000i/SP ion chromatograph that contained an ion Pac AS4A column (Dionex GmbH, Idstein, Germany). uPRAL was calculated as follows:

uPRAL = Chloride (mmol/d) + sulfate (mmol/d) x 2 + phosphate (mmol/d) x 1.8 – sodium (mmol/d) – potassium (mmol/d) – magnesium (mmol/d) x 2 – calcium (mmol/d) x 2)^24^. The ion excretion in mmol/d is converted to milliequivalents per day (mEq/d) by multiplying with the respective ionic valence^25^.

## Blood measurements

Venous blood samples (< 20 ml) were collected after an overnight fast, promptly centrifuged at 4 °C within 15 min, and stored at -80 °C. Routine blood parameters such as glucose or blood lipids were quantified immediately after blood withdrawal. Mean storage duration of plasma samples of participants with the earliest adulthood blood collections included, amounted to 9.4 ± 5.1 years until FGF23 and α-klotho analyses. Plasma concentrations of FGF23, α-klotho, PTH, and insulin were measured at the Laboratory for Translational Hormone Analytics of the University of Giessen. For FGF23 analysis the sandwich enzyme immunoassay kit FGF23 ELISA (EIA-6060; DRG Diagnostics, Marburg, Germany) was used, which detects and binds to an epitope within the carboxyl-terminal (C-Term) portion of the FGF23 molecule. The human soluble α-klotho sandwich ELISA kit (IBL No. 27998; Immuno-Biological Laboratories Co., Ltd, Gunma, Japan) was used for the quantification of α-klotho. PTH was analyzed by the two-site Parathyroid hormone intact ELISA (NM 59041; IBL International GmbH, Hamburg, Germany). Plasma insulin concentrations were quantified with an immunoradiometric assay (IRMA; DRG Diagnostics, Marburg, Germany) and the homeostasis model assessment was used to evaluate insulin resistance (HOMA-IR)^26^.

Serum concentrations of glucose, LDL, and HDL cholesterol as well as plasma concentrations of total calcium, albumin, and phosphate were measured with a Roche/Hitachi Cobas c311 analyser (Roche diagnostics, Mannheim, Germany) at the clinic lab of the pediatric clinic Dortmund, Germany. Albumin-corrected calcium was determined as a surrogate parameter for ionized calcium concentration using the Parfitt formula: corrected calcium = total calcium (mmol/L) + [(40 – albumin (g/L) /40]^27^.

The estimated glomerular filtration rate (eGFR) was calculated according to the European Kidney Function Consortium (EKFC) Eqs^28,29^. In 264 of the included 343 study participants, also circulating leptin, adiponectin, soluble intercellular adhesion molecule-1 (sICAM-1), omentin, interleukin (IL)-1 receptor antagonist (IL-1RA), fetuin-A, IL-18, IL-6, soluble E-selectin (sE-selectin), and high-sensitivity C-reactive protein (hsCRP) were determined at the Institute for Clinical Diabetology (German Diabetes Center, Düsseldorf, Germany) using highly sensitive ELISAs^30^ and a Roche/Hitachi Cobas c311 analyser (Roche diagnostics, Mannheim, Germany) for hsCRP.

### Statistical analysis

All statistical analyses were performed using SAS statistical software (SAS Institute Inc., Cary, NC, USA; version 9.2), and statistical tests were considered significant if P-values were < 0.05. Normal distribution of all variables was checked through Shapiro-Wilk test and Q-Q plot.

Descriptive data of the participants are shown as mean (± SD) for normally distributed parameters and as median (25th, 75th percentiles) for non-normally distributed. Differences between the first and the last assessment (Table 1) were tested using paired t-test for normally distributed data, and Wilcoxon signed-rank test for non-normally distributed characteristics. IL-6, α-klotho and HOMA-IR were log_10_ transformed in all regression models to normalize their distribution.

**Table 1:** Characteristics of participants during growth and adulthood. All values are means ± SDs if normally distributed or median (25th, 75th percentiles) if not normally distributed. BMI, body mass index; BSA, body surface area; eGFR, estimated glomerular filtration rate; FGF23, fibroblast growth factor 23; FMI, fat mass index; HDL, high density lipoprotein cholesterol; HOMA-IR, homeostasis model assessment-insulin resistance; IL-6, interleukin-6; LDL, low density lipoprotein cholesterol; NAE, net acid excretion; PO4, phosphate; PTH, parathyroid hormone; uPRAL, urinary potential renal acid load; Urea-N, 24 h urinary urea nitrogen excretion. ^a^The first and the last urine sample of each participant who had at least 2 eligible urine samples collected between age 3 and 8 y, and at least 3 eligible urine samples collected between age 9–17 y (*n* = 343; average number of urines collected per person = 9.8). ^b^Differences between the first and last assessments were tested with paired *t* test for normally distributed variables and Wilcoxon signed rank test for non-normally distributed ones (all differences *P* < 0.0001, except 24-h urinary pH). ^c^The reference range for normal adult values of FGF23 according to the assay producer: 0–20 pmol/L. ^d^Calcium levels corrected for serum albumin concentration. ^e^GFR assessed using the EKFC equations. GFR was 107.7 ± 19.2 mL/min/1.73 m² if assessed using the creatinine-based equations of the chronic kidney disease epidemiology collaboration (CKD-EPI)^52^. ^f^Of all proinflammatory markers checked (see methods), only IL-6 measurements fulfilled the regression model criteria and was finally included in the regression model. IL-6 measurements were available for 264 participants in adulthood. ^g^Subject number: *n* = 210, analyte measurements for uPRAL determination were available for 108 males and 102 females. 133 participants did not collect a 24-h urine sample at the date of blood sampling. ^h^Subject number: *n* = 94 (50 males and 44 females) NAE measurements were performed only sporadically in adults.

Childhood & adolescence	Longitudinal overview^a^	
	First assessment	Last assessment^b^
n (male/female)	343 (174/169)	343 (174/169)
Age, y	4.0 (3.4, 5.0)	16.1 (14.0, 17.0)
Weight, kg	18.0 ± 3.5	60.8 ± 16.0
Height, cm	107.0 ± 8.4	169.3 ± 13.7
BMI, kg/m²	15.6 ± 1.2	20.8 ± 3.5
BSA, m²	0.7 ± 0.1	1.7 ± 0.3
FMI, kg/m²	2.5 ± 0.7	5.0 ± 2.8
Urine, pH	6.4 ± 0.5	6.3 ± 0.5
Urea-N, mmol/d	168.9 ± 52.8	341.0 ± 115.6
Ca, mmol/d	0.80 (0.5, 1.3)	2.6 (1.5, 3.9)
Na, mmol/d	58.4 ± 24.1	128.2 ± 56.5
Cl, mmol/d	58.3 ± 22.9	129.6 ± 55.5
Osmolality, mOsm/d	349.1 ± 95.5	714.6 ± 221.2
PO4, mmol/d	13.5 ± 4.1	25.7 ± 8.8
PO4 (mmol/d/1.73 m²)	32.2 ± 8.4	26.1 ± 7.2
NAE, mEq/d	23.7 ± 12.3	52.0 ± 25.8
NAE (mEq/d/1.73 m²)	56.1 ± 27.3	52.6 ± 23.0
uPRAL, mEq/d	2.8 ± 13.7	16.6 ± 23.4
uPRAL (mEq/d/1.73 m²)	6.1 ± 32.9	16.7 ± 22.5
**Adulthood**		
Age, y	20.8 (18.0, 23.5)	
Weight, kg	73.0 ± 16.0	
Height, cm	176.9 ± 9.4	
BMI, kg/m²	23.2 ± 4.1	
FGF23 (pmol/L)^c^	1.4 ± 0.9	
α-klotho (pg/ml)	397.7 (296.9, 541.2)	
PO4 (mmol/L)	1.08 ± 0.1	
Albumin (g/L)	44.6 ± 3.0	
Ca (mmol/L)	2.2 ± 0.9	
Ca-albumin corrected^d^	2.1 ± 0.8	
PTH (pg/ml)	50.9 ± 20.7	
HOMA-IR	2.5 (1.9, 3.2)	
LDL (mg/dl)	96.4 ± 29.8	
HDL (mg/dl)	58.0 ± 15.0	
Uric acid (mg/dl)	5.2 ± 1.2	
Creatinine (mg/dl)	0.9 ± 0.2	
GFR (mL/min/1.73 m²) ^e^	93.6 (80.7, 107.3)	
IL-6 (pg/ml)^f^	0.6 (0.4, 1.0)	
uPRAL, mEq/d ^g^	18.6 ± 31.9	
uPRAL (mEq/d/1.73 m²)	16.3 ± 27.6	
NAE, mEq/d ^h^	62.7 ± 36.5	
NAE (mEq/d/1.73 m²)	56.6 ± 28.6	

Anthropometric assessments and all pre-adulthood 24-h urinary biomarkers were internally standardized (mean = 0, SD = 1) by sex and age and the standardized deviation scores (SDS) were averaged as means of all individually available measurement points for each participant. Anthropometric and 24-h urinary excretion data of the growth period were then included in the analyses as the respective individual arithmetic means of SDS.

To examine the prospective associations of long-term higher phosphorus intake (assessed by PO4 excretion), higher net endogenous acid production (NAE), and higher urinary potential renal acid load (uPRAL) in childhood and adolescence with the adulthood outcomes fibroblast growth factor-23 (FGF23) and α-klotho, multiple linear regression models were performed (PROC GLM in SAS). The assumptions of multi-linear regressions, i.e., normal distribution of residuals, linearity, non-multicollinearity, and homoscedasticity were tested and not violated. Regression analyses were conducted without sex stratification since sex-specific interactions were not observed (*P >* 0.1).

In order to be included in the final statistical model, each potential covariate was tested separately by the use of stepwise regression. Covariates were included in the regression model if: (i) they modified the association between the exposition variables PO4 excretion, NAE, or uPRAL and the outcomes FGF23 or α-klotho (i.e., if changes in the β coefficient of predictors were ≥ 10%), (ii) they had an independent and significant effect on the outcome FGF23 (*P* < 0.05), or (iii) they improved the explained variability of the model.

A complementary multiple linear regression analysis was performed using phosphate excretion as the independent variable and the FGF23/α-klotho ratio as the dependent variable (Supplementary table S1). A sensitivity analysis was conducted in a reduced dataset of participants in whom also blood levels of biomarkers of inflammation had been analyzed. In the final sensitivity analysis only IL-6 was additionally allowed for, as among the ten measured biomarkers of inflammation only IL-6 did show a significant relationship with FGF23, without any further association with α-klotho (Supplementary table S2).

## Results

Table 1 provides an overview of the anthropometric, blood and 24-h urinary excretion characteristics of the study participants at their first and last assessment. The median age of the 343 individuals (174 males and 169 females) was 4 years at the first assessment and 16 years at the last assessment. Children’s and adolescents’ absolute 24-h urinary PO4-Ex, NAE and PRAL markedly increased between the initial and final evaluations (*p*< 0.0001). Corrected for an adult body surface area (BSA) of 1.73m^2^, PO4-Ex corresponded to 32 mmol/d/1.73m^2^ at baseline and 26 mmol/d/1.73m^2^ in adolescence. Net acid excretion (NAE) and urinary potential renal acid load (uPRAL) varied from 56 mEq/d/1.73m^2^ and 6 mEq/d/1.73m^2^, respectively, in the early years to 52 mEq/d/1.73m^2^ and 17 mEq/d/1.73m^2^ at the last urine collection. Participants’ urinary pH fluctuated around ~ 6.4 with a slight decrease at the last evaluation (*p* = 0.01). All other parameters (anthropometric as well as 24-h urinary excretion rates), except NAE related to BSA, significantly (*p* < 0.0001) differed between the two assessments. FGF23 and α-klotho levels measured in adults’ fasting blood samples were 1.4 pmol/L and 397.7 pg/ml, respectively.

Partial correlations examining the outcomes FG23 and α-klotho in relation to the exposures 24-h urinary PO4-Ex, NAE and uPRAL are presented in Table 2. After adjustment of outcomes and exposures for age and urea nitrogen excretion, respectively, FGF23, but not α-klotho, demonstrated a positive and statistically significant correlation exclusively with PO4-Ex (Table 2; *r* = 0.14, *p* = 0.008).

**Table 2:** Partial correlations of adult circulating FGF23 and α-klotho with urinary 24-h excretion SDS of the pre-adulthood exposures phosphate (PO4) excretion, net acid excretion (NAE), and urinary potential renal acid load (PRAL). ^a^ FGF23 and α-klotho both adjusted for adult age. ^b^ 24-h urinary biomarker SDS each adjusted for 24-h urea nitrogen excretion SDS.

	FGF23 ^a^ (pmol/L)		α-klotho ^a^ (pg/ml)	
	*r*	*p*	*r*	*p*
PO4-SDS (mmol/d) ^b^	0.14	0.008	-0.04	0.43
NAE-SDS (mEq/d) ^b^	0.09	0.10	-0.04	0.48
uPRAL-SDS (mEq/d) ^b^	-0.009	0.87	0.008	0.88

**Fig. 1:**
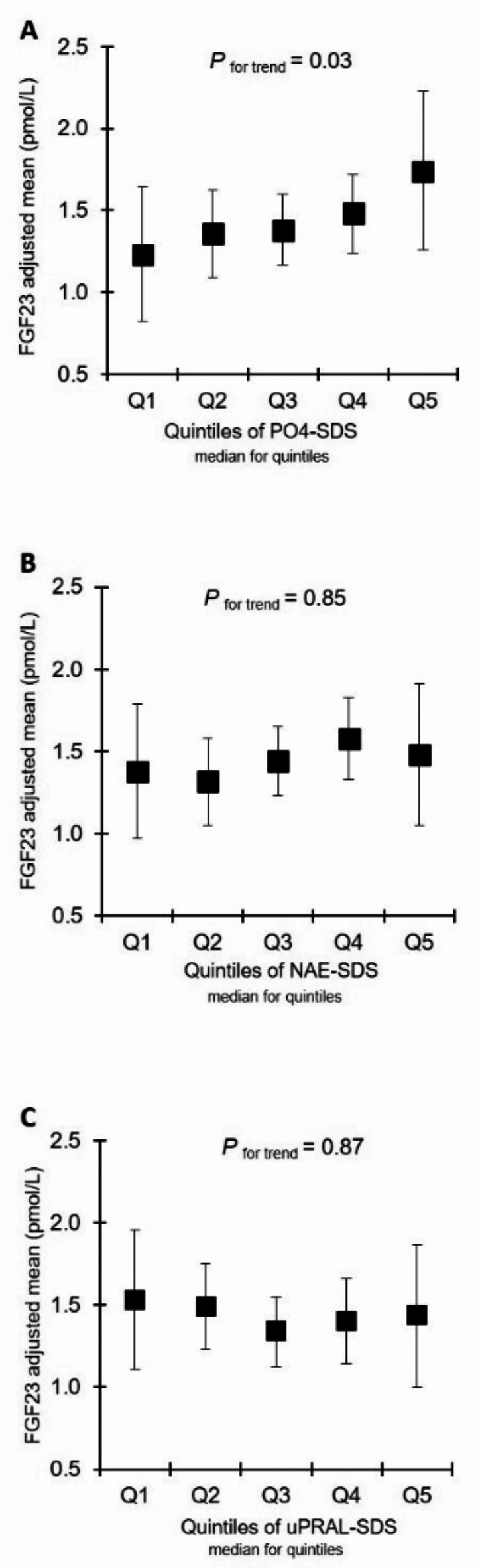
Adjusted means (95% CI) of young adults´ circulating FGF23 in quintiles of phosphate excretion SDS (**A**), net acid excretion SDS (**B**), and urinary potential renal acid load SDS (**C**) during growth. SDS-excretion of each quintile represent the median of the respective quintile. Adjusted means were derived from multiple linear regression models after adjustment for the covariates sex, adults’ age, mean SDS of children´s and adolescents´ nutrition-related 24-h urinary biomarkers (urea nitrogen, osmolality, calcium, salt and pH), and adults’ blood-derived-parameters (PTH, HOMA-IR, LDL/HDL ratio, uric acid and phosphate). P values were obtained from the linear regression models. Q, quantiles; NAE, renal net acid excretion; uPRAL, urinary potential renal acid load; SDS, standard deviation score.

**Fig. 2:**
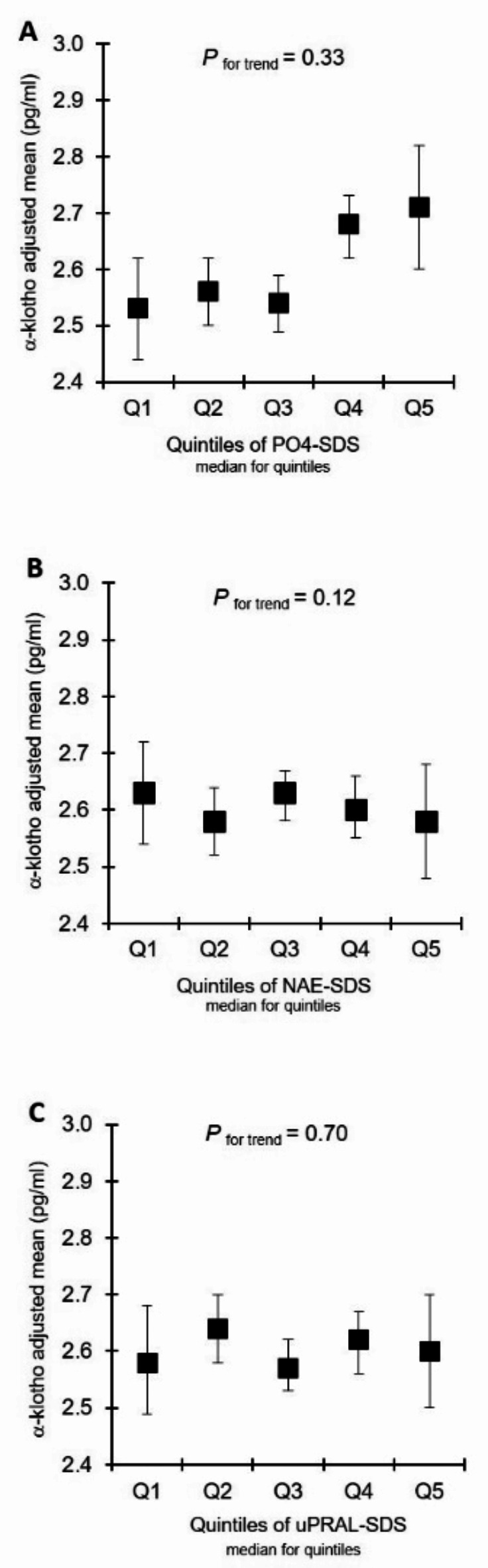
Adjusted means (95% CI) of young adults´ soluble α-klotho in quintiles of phosphate excretion SDS (**A**), net acid excretion SDS (**B**), and urinary potential renal acid load SDS (**C**) during growth. SDS-excretion of each quintile represent the median of the respective quintile. Adjusted means were derived from multiple linear regression models after adjustment for the covariates sex, adults’ age, adults’ BMI, mean SDS of children and adolescents nutrition-related 24-h urinary biomarkers (urea nitrogen, osmolality, and calcium) and adults’ blood-derived-parameters (PTH, LDL/HDL ratio, albumin, GFR and phosphate) (**A**); and additionally 24-h urinary pH (**B**). In diagram **C** (uPRAL) sex, adults’ age, adults’ BMI, 24-h urinary pH and sodium excretion as well as PTH, LDL/HDL ratio, uric acid and phosphate were adjusted for. P values were obtained from the linear regression models. Q, quantiles; NAE, renal net acid excretion; uPRAL, urinary potential renal acid load; SDS, standard deviation score.

The fully adjusted relationships of adult circulating FGF23 and α-klotho with pre-adulthood PO4-Ex and habitual dietary acid load (NAE and uPRAL) are shown as adjusted means (with 95% confidence intervals) in quintiles of children’s and adolescents’ median values of SDS of PO4-Ex, NAE and uPRAL (Figs. 1 and 2). A significant association was only observable for FGF23 and preceding PO4-Ex during growth. No such association was discernible between FGF23 and parameters related to habitual dietary acid load. For circulating α-klotho no significant association was found with any of the exposures.

Estimated glomerular filtration rate (eGFR) was considered in all regression analyses using the stepwise selection criteria, but it only fulfills the inclusion criteria in the α-klotho regression model. Supplementary Figure S1 demonstrates the lack of association between eGFR and the outcomes FGF23 and α-klotho in young healthy adults, which contrasts the situation in patients with lower GFR or chronic kidney disease^31^.

Also when the ratio of FGF23/klotho, as a potential indicator of FGF23 resistance, was analyzed regarding possible associations with the exposures no significant result was observed. However, a trend (*p* = 0.10) was discernible for the association of the biomarker of long-term phosphorus intake with the FGF23/klotho ratio (Supplementary table S1).

In the sensitivity analysis in the reduced sample subset including IL-6 as an additional covariate, the aforementioned study results regarding long-term higher phosphorus intake and increased FGF23 levels were confirmed (Supplementary table S2).

## Discussion

This study examined, renal biomarker-based, habitual phosphorus intake (P-In) and habitual dietary acid load of healthy children and adolescents and their relationships with blood levels of FGF23 and α-klotho later in adulthood. Our findings suggest that higher P-In in the growing years may unfavorably affect adults´ endocrine FGF23–α-klotho axis via raised FGF23, without parallel increase of α-klotho.

The significant relationship between FGF23 and 24-h PO4-excretion levels – observed after specifically adjusting for adults´ blood phosphate concentrations, insulin resistance state, circulating calcium, PTH, blood lipids, and the probands’ habitual protein and salt intake during growth – could be further substantiated by a sensitivity analysis. This sensitivity analysis, run in a reduced sample subset, additionally considered pro-inflammatory markers and was specifically adjusted for IL-6. Also the FGF23/klotho ratio, i.e., an indicator for FGF23 resistance, associated – although not significant – with the children’s and adolescents´ PO4-excretion rates, whereas circulating α-klotho itself did not show an association. In contrast to the biomarker for P-In during growth, renal biomarkers for habitual dietary acid load (24-h NAE or 24-h uPRAL) did not associate with adult circulating FGF23, α-klotho, or the FGF23/klotho ratio.

Accordingly, the present longitudinal biomarker-based prospective findings suggest that an incipient adult FGF23 resistance may be related to even just a habitually higher phosphorus intake still in the normal range of healthy children and youth.

Within the proximal tubules, both transmembrane klotho and soluble klotho facilitate the binding of FGF23 to the FGF receptors and hence induce the removal of NaPI-IIa and NaPi-IIc transporters from the apical membrane. This process reduces phosphate reabsorption and promotes urinary phosphate excretion, thus impacting the systemic phosphate balance^32^.

A decline in the levels of circulating soluble α-klotho has been noted during the initial stages of chronic kidney disease (CKD), indicating renal function loss^33^. As kidney function declines, serum phosphate levels increase, triggering the secretion of phosphaturic FGF23^34^, of which elevated levels associate with cardiovascular and mortality risks^34,35^.

In healthy adults, short-term high dietary or intravenous phosphate loads significantly raise FGF23 blood levels^8,9,36^, whereas low to moderate increases in phosphate administration do not consistently increase FGF23^37,38^. In a cross-sectional observational study in healthy children on normal varying diets, no association has been found between FGF23 and phosphate concentrations in serum or spot urine samples^11^. Our study in healthy children and youth adds specific and substantially new data to the hitherto inconsistent findings on children’s FGF23–P-In relationship in that a habitual high P-In with usual childhood and adolescent nutrition has been shown to affect the FGF23–α-klotho axis.

Children’s and adolescents´ regular P-In exceeds Recommended Dietary Allowances^39^in around 50% of the study participants^12^. For α-klotho, no relationship with any of the pre-adulthood exposition variables was discernible. Studies that experimentally raised phosphate loads in healthy people reported inconsistent results with either α-klotho rises that paralleled the FGF23 rises^40^or without klotho increases^8^. In chronic kidney disease (CKD) and even in the early stages of this illness α-klotho levels decrease along with increases in FGF23^41,42^.

Complex interactions exist between FGF23, kidney function, and endogenous acid production ensuring systemic electrolyte balance. Independent from the hormonal effects of FGF23 and PTH on renal phosphate balance, the proximal tubular phosphate reabsorption immediately drops if free protons increase after an increase in acid loading^15^. Hence, the additional phosphate anions in the tubule lumen can directly attach excess protons, forming titratable acidity and serving as a urinary buffer^43^. In an intervention study in healthy adult females, the administration of ammonium chloride for 7 days, resulting in chronic metabolic acidosis, led to an elevated fractional phosphate excretion, consequent hypophosphatemia, and hence a reduction in FGF23 plasma levels^14^. Lowered plasma FGF23 levels were also observed in healthy persons after switching from a low-protein to an acidic high protein diet in a dietary modification trial^44^.

In the present study no association between FGF23 and habitual dietary acid load, neither for uPRAL nor for NAE was seen. It remains unclear whether the failure to detect any FGF23 relationship with long-term endogenous acid production might have been due to habitual dietary acid loads that did not vary appropriately. However, it appears rather clear that the elevated FGF23 levels frequently seen in CKD patients with metabolic acidosis^45^are not primarily caused by the acidosis itself, but may rather be due to other factors disturbing and stimulating the FGF23/klotho system in CKD^46,47^. Accordingly, also in CKD patients examined in the Chronic Renal Insufficiency (CRIC) Cohort Study, neither NAE nor PRAL was associated with FGF23^48^.

Numerous experimental studies have revealed that pro-inflammatory cytokines, including IL-6, can dose-dependently upregulate FGF23 gene expression and stimulate the synthesis of FGF23^35,47^. Accordingly, the sensitivity analysis performed in the reduced data set of those study participants in whom also pro-inflammatory cytokines had been measured, confirmed not only an increase in overall explained variance of the model after including IL-6, but also the independent relationship between long-term higher phosphorus intake and FGF23 (Supplementary Table S2. model IV).

Due to the exclusively noninvasive character of the DONALD study throughout the entire growth period, no childhood baseline data on the FGF23–α-klotho axis, pro-inflammatory cytokines, and further relevant metabolic blood parameters were available. Another limitation is that 24-h urine collections, which would allow to determine PO4, NAE, and uPRAL when FGF23 and a-klotho were assessed, were only performed in a markedly reduced subset of participants. Also, we were not able to differentiate between natural phosphorus intake from food and phosphate salts added to food. This differentiation might have let to even clearer results, since particularly the consumption of inorganic phosphate food additives, which have a markedly higher bioavailability than natural phosphorus^49^, can lead to more pronounced rises in blood PO4 concentrations^50^. As a majority of studies on FGF23 were performed by the use of the C-terminal FGF23 assay (cFGF23)^51^and some evidence reported that cFGF23 outperforms the intact FGF23 assay in predicting decline in renal function^51^, we opted for using cFGF23. The cFGF23 measures both the intact molecule and the large carboxyl terminal fragment of human FGF23, partially in vivo cleaved from the synthesized intact FGF23, whereas the intact FGF23 assay only detects the whole molecule. The performance characteristics of both assay types show limitations and vary widely in detection limits, measurement ranges and precision. Accordingly, the additional quantification also of the full-length intact FGF23 may have provided supplementary information on the long-term relevance of high P-In in the growing years for young adults’ FGF23-α-klotho axis. Furthermore, it has to be mentioned that the present findings, as obtained in a cohort of children of European descent, are not necessarily applicable to other ethnicities.

Notwithstanding these limitations, the strength of the current study lies in its detailed data and sample collection, involving repeated 24-hour urine measurements over a long period, which enables an adequate control of interacting covariates and the specific adjustment of an inherent confounder of phosphorus ingestion i.e., dietary protein intake, as has been accomplished through adjusting for the biomarker urinary urea nitrogen excretion. Moreover, the gold standard method for determining net endogenous acid production was employed to assess the long-term dietary acid load of the children and adolescents. But even more important, we could adjust for the blood phosphate concentration that directly determines circulating FGF23, so that a confounding effect through an acute high P-In can – at the time of blood collection – be at least partly excluded.

Overall, our findings contribute to an improved understanding of the different factors influencing the FGF23 dynamics from childhood onward in the long-run and provide evidence that higher phosphorus intake, but not habitually higher net endogenous acid production of children and youth may be associated with the endocrine FGF23–α-klotho axis later in adulthood. It is concluded that a regularly high phosphorus intake during the growing years may raise the set point of FGF23, without distinctly altering α-klotho, implying that the risk for potential harmful FGF23 actions on cardiovascular health or bone mineralization may already become triggered through an unhealthy phosphorus-rich nutrition in childhood and adolescence.

## Electronic supplementary material

Below is the link to the electronic supplementary material.

Supplementary Material 1

Supplementary Material 2

Supplementary Material 3

## Data Availability

Data described in the manuscript will be made available upon request pending application to the corresponding author and approval by the Institute of Nutrition and Food Sciences, Nutritional Epidemiology, University of Bonn, Bonn, Germany.

## References

[1] Fukumoto, S. FGF23-related hypophosphatemic rickets/osteomalacia: diagnosis and new treatment. *J. Mol. Endocrinol.* **66**10.1530/JME-20-0089 (2021). 10.1530/JME-20-0089 33295878

[2] Wagner, C. A. The basics of phosphate metabolism. *Nephrology, dialysis, transplantation: official publication of the European Dialysis and Transplant Association -*. *Eur. Ren. Association*. **39**, 190–201. 10.1093/ndt/gfad188 (2024). 10.1093/ndt/gfad188 PMC10828206 37660247

[3] Salcedo-Betancourt, J. D. & Moe, O. W. The effects of Acid on calcium and phosphate metabolism. *Int. J. Mol. Sci.* **25**10.3390/ijms25042081 (2024). 10.3390/ijms25042081 PMC10889523 38396761

[4] Hu, M. C., Shi, M. & Moe, O. W. Role of αKlotho and FGF23 in regulation of type II Na-dependent phosphate co-transporters. *Pflug, Arch.: Eur. J. Physiol.* **471**, 99–108. 10.1007/s00424-018-2238-5 (2019). 10.1007/s00424-018-2238-5 PMC6324980 30506274

[5] Kuro-O, M. & Moe, O. W. FGF23-αKlotho as a paradigm for a kidney-bone network. *Bone*. **100**, 4–18. 10.1016/j.bone.2016.11.013 (2017). 27847255 10.1016/j.bone.2016.11.013

[6] Li, Y. et al. Klotho is regulated by transcription factor Sp1 in renal tubular epithelial cells. *BMC Mol. cell. Biology*. **21**10.1186/s12860-020-00292-z (2020). 10.1186/s12860-020-00292-z PMC7309980 32571212

[7] Castner, S. A. et al. Longevity factor klotho enhances cognition in aged nonhuman primates. *Nat. Aging*. **3**, 931–937. 10.1038/s43587-023-00441-x (2023). 37400721 10.1038/s43587-023-00441-x PMC10432271

[8] Scanni, R., vonRotz, M., Jehle, S., Hulter, H. N. & Krapf, R. The human response to acute enteral and parenteral phosphate loads. *J. Am. Soc. Nephrology: JASN*. **25**, 2730–2739. 10.1681/ASN.2013101076 (2014). 10.1681/ASN.2013101076 PMC4243350 24854273

[9] Vervloet, M. G. et al. Effects of dietary phosphate and calcium intake on fibroblast growth factor-23. *Clin. J. Am. Soc. Nephrology: CJASN*. **6**, 383–389. 10.2215/CJN.04730510 (2011). 10.2215/CJN.04730510 PMC3052230 21030580

[10] Ito, N. et al. Effect of acute changes of serum phosphate on fibroblast growth factor (FGF)23 levels in humans. *J. Bone Miner. Metab.* **25**, 419–422. 10.1007/s00774-007-0779-3 (2007). 17968495 10.1007/s00774-007-0779-3

[11] Mitchell, D. M., Jüppner, H. & Burnett-Bowie, S. A. M. FGF23 is not Associated with Age-related changes in phosphate, but enhances renal calcium reabsorption in girls. *J. Clin. Endocrinol. Metab.* **102**, 1151–1160. https://doi.org/10.1210/jc.2016–4038 (2017). 10.1210/jc.2016-4038 PMC5460726 28323960

[12] Franco, L. et al. Phosphorus intake and potential dietary influences examined via 24-Hour urinary biomarker measurements in German children and adolescents over 3 decades. *J. Acad. Nutr. Dietetics*. 10.1016/j.jand.2024.02.008 (2024). 10.1016/j.jand.2024.02.008 38360183

[13] Fayed, A. et al. Fibroblast growth factor-23 is a strong predictor of insulin resistance among chronic kidney disease patients. *Ren. Fail.* **40**, 226–230. 10.1080/0886022X.2018.1455594 (2018). 29619868 10.1080/0886022X.2018.1455594 PMC6014287

[14] Domrongkitchaiporn, S. et al. Oral phosphate supplementation corrects hypophosphatemia and normalizes plasma FGF23 and 25-hydroxyvitamin D3 levels in women with chronic metabolic acidosis. *Experimental and clinical endocrinology & diabetes: official journal*. *German Soc. Endocrinol. [and] German Diabetes Association*. **118**, 105–112. 10.1055/s-0029-1202791 (2010). 10.1055/s-0029-1202791 19449283

[15] Nowik, M. et al. Renal phosphaturia during metabolic acidosis revisited: molecular mechanisms for decreased renal phosphate reabsorption. *Pflug, Arch.: Eur. J. Physiol.* **457**, 539–549. 10.1007/s00424-008-0530-5 (2008). 10.1007/s00424-008-0530-5 18535837

[16] Perrar, I., Alexy, U. & Nöthlings, U. Cohort profile update-overview of over 35 years of research in the Dortmund Nutritional and Anthropometric longitudinally designed (DONALD) study. *Eur. J. Nutr.* **63**, 727–740. 10.1007/s00394-023-03290-x (2024). 38151532 10.1007/s00394-023-03290-x PMC10948456

[17] Kroke, A. et al. The DONALD Study. History, current status and future perspectives. *Eur. J. Nutr.* **43**, 45–54. 10.1007/s00394-004-0445-7 (2004). 14991269 10.1007/s00394-004-0445-7

[18] Buyken, A. E., Alexy, U., Kersting, M., Remer, T. & Die, D. O. N. A. L. D. Kohorte. Ein Aktueller Überblick zu 25 Jahren Forschung Im Rahmen Der Dortmund Nutritional and Anthropometric longitudinally designed study. *Bundesgesundheitsblatt Gesundheitsforschung Gesundheitsschutz*. **55**, 875–884. 10.1007/s00103-012-1503-6 (2012). 22736170 10.1007/s00103-012-1503-6

[19] Du Bois, D. & Du Bois, E. F. A formula to estimate the approximate surface area if height and weight be known. *Nutrition (Burbank, Los Angeles County, Calif.)* 5, 303 – 11; discussion 312-3 (1989). (1916). 2520314

[20] Slaughter, M. H. et al. Skinfold equations for estimation of body fatness in children and youth. *Hum. Biol.* **60**, 709–723 (1988). 3224965

[21] Remer, T., Montenegro-Bethancourt, G. & Shi, L. Long-term urine biobanking: storage stability of clinical chemical parameters under moderate freezing conditions without use of preservatives. *Clin. Biochem.* **47**, 307–311. 10.1016/j.clinbiochem.2014.09.009 (2014). 25239781 10.1016/j.clinbiochem.2014.09.009

[22] Remer, T., Neubert, A. & Maser-Gluth, C. Anthropometry-based reference values for 24-h urinary creatinine excretion during growth and their use in endocrine and nutritional research. *Am. J. Clin. Nutr.* **75**, 561–569. 10.1093/ajcn/75.3.561 (2002). 11864864 10.1093/ajcn/75.3.561

[23] Lüthy, C., Moser, C. & Oetliker, O. Dreistufige Säure-Basen-Titration Im Urin. *Das Medizinische Laboratorium*. **30**, 174–181 (1977). 20563

[24] Remer, T., Dimitriou, T. & Manz, F. Dietary potential renal acid load and renal net acid excretion in healthy, free-living children and adolescents. *Am. J. Clin. Nutr.* **77**, 1255–1260. 10.1093/ajcn/77.5.1255 (2003). 12716680 10.1093/ajcn/77.5.1255

[25] Esche, J., Johner, S., Shi, L., Schönau, E. & Remer, T. Urinary citrate, an index of acid-base status, predicts bone strength in youths and fracture risk in adult females. *J. Clin. Endocrinol. Metab.* **101**, 4914–4921. https://doi.org/10.1210/jc.2016–2677 (2016). 10.1210/jc.2016-2677 27676395

[26] Wallace, T. M., Levy, J. C. & Matthews, D. R. Use and abuse of HOMA modeling. *Diabetes care*. **27**, 1487–1495. 10.2337/diacare.27.6.1487 (2004). 15161807 10.2337/diacare.27.6.1487

[27] Centre Suisse de Contrôle de Qualité (CSCQ). Leistungsdokument Nr. 23. Bestimmung von Kalzium und Phosphat. CSCQ, 2 chemin du Petit-Bel-Air, CH – 1225 Chêne-Bourg, März (2009).

[28] Delanaye, P. et al. Estimating glomerular filtration in young people. *Clinical kidney journal* 17, sfae261; (2024). 10.1093/ckj/sfae261 10.1093/ckj/sfae261 PMC11418036 39314869

[29] Delanaye, P., Cavalier, E., Pottel, H. & Stehlé, T. New and old GFR equations: a European perspective. *Clin. Kidney J.* **16**, 1375–1383. 10.1093/ckj/sfad039 (2023). 37664574 10.1093/ckj/sfad039 PMC10469124

[30] Hua, Y. et al. Inflammatory mediators in the adipo-renal axis: leptin, adiponectin, and soluble ICAM-1. *Am. J. Physiol. Renal. Physiol.* **319**10.1152/ajprenal.00257.2020 (2020). F469-F475. 10.1152/ajprenal.00257.2020 32744085

[31] Verzola, D. et al. Interorgan handling of fibroblast growth factor-23 in humans. *Am. J. Physiol. Renal. Physiol.* **312**10.1152/ajprenal.00396.2016 (2017). F254-F258. 10.1152/ajprenal.00396.2016 27558560

[32] Frassetto, L. A., Sebastian, A. & DuBose, T. D. How metabolic acidosis and kidney disease may accelerate the aging process. *Eur. J. Clin. Nutr.* **74**, 27–32. 10.1038/s41430-020-0693-5 (2020). 32873954 10.1038/s41430-020-0693-5

[33] Jacquillet, G. & Unwin, R. J. Physiological regulation of phosphate by vitamin D, parathyroid hormone (PTH) and phosphate (pi). *Pflug, Arch.: Eur. J. Physiol.* **471**, 83–98. 10.1007/s00424-018-2231-z (2019). 10.1007/s00424-018-2231-z PMC6326012 30393837

[34] Vogt, I., Haffner, D. & Leifheit-Nestler, M. FGF23 and phosphate-Cardiovascular Toxins in CKD. *Toxins*. **11**10.3390/toxins11110647 (2019). 10.3390/toxins11110647 PMC6891626 31698866

[35] Durlacher-Betzer, K. et al. Interleukin-6 contributes to the increase in fibroblast growth factor 23 expression in acute and chronic kidney disease. *Kidney Int.* **94**, 315–325. 10.1016/j.kint.2018.02.026 (2018). 29861060 10.1016/j.kint.2018.02.026

[36] Ferrari, S. L., Bonjour, J. P. & Rizzoli, R. Fibroblast growth factor-23 relationship to dietary phosphate and renal phosphate handling in healthy young men. *J. Clin. Endocrinol. Metab.* **90**, 1519–1524 (2005). 10.1210/jc.2004 – 1039. 15613425 10.1210/jc.2004-1039

[37] Nishida, Y. et al. Acute effect of oral phosphate loading on serum fibroblast growth factor 23 levels in healthy men. *Kidney Int.* **70**, 2141–2147. 10.1038/sj.ki.5002000 (2006). 17063170 10.1038/sj.ki.5002000

[38] Irzyniec, T. et al. The effect of an oral sodium phosphate load on parathyroid hormone and fibroblast growth factor 23 secretion in normo- and hypercalciuric stone-forming patients. *Clin. Nutr.* **39**, 3804–3812. 10.1016/j.clnu.2020.04.020 (2020). 32386861 10.1016/j.clnu.2020.04.020

[39] *Dietary Reference Intakes for Calcium, Phosphorus, Magnesium, Vitamin D, and Fluoride* (Washington (DC), (1997). 23115811

[40] Mohammad, J., Scanni, R., Bestmann, L., Hulter, H. N. & Krapf, R. A controlled increase in dietary phosphate elevates BP in healthy human subjects. *J. Am. Soc. Nephrology: JASN*. **29**, 2089–2098. 10.1681/ASN.2017121254 (2018). 10.1681/ASN.2017121254 PMC6065082 30021759

[41] Edmonston, D., Grabner, A. & Wolf, M. FGF23 and klotho at the intersection of kidney and cardiovascular disease. *Nat. Rev. Cardiol.* **21**, 11–24. 10.1038/s41569-023-00903-0 (2024). 37443358 10.1038/s41569-023-00903-0

[42] Shimamura, Y. et al. Serum levels of soluble secreted α-Klotho are decreased in the early stages of chronic kidney disease, making it a probable novel biomarker for early diagnosis. *Clin. Exp. Nephrol.* **16**, 722–729. 10.1007/s10157-012-0621-7 (2012). 22457086 10.1007/s10157-012-0621-7

[43] Wesson, D. E., Buysse, J. M. & Bushinsky, D. A. Mechanisms of Metabolic Acidosis-Induced kidney Injury in chronic kidney disease. *J. Am. Soc. Nephrology: JASN*. **31**, 469–482. 10.1681/ASN.2019070677 (2020). 10.1681/ASN.2019070677 PMC7062220 31988269

[44] Kremsdorf, R. A. et al. Effects of a high-protein diet on regulation of phosphorus homeostasis. *J. Clin. Endocrinol. Metab.* **98**, 1207–1213. https://doi.org/10.1210/jc.2012–2910 (2013). 10.1210/jc.2012-2910 PMC3590482 23393178

[45] Silver, J. & Naveh-Many, T. FGF-23 and secondary hyperparathyroidism in chronic kidney disease. *Nat. Rev. Nephrol.* **9**, 641–649. 10.1038/nrneph.2013.147 (2013). 23877588 10.1038/nrneph.2013.147

[46] Mace, M. L., Olgaard, K. & Lewin, E. New aspects of the kidney in the regulation of fibroblast growth factor 23 (FGF23) and Mineral Homeostasis. *Int. J. Mol. Sci.* **21**10.3390/ijms21228810 (2020). 10.3390/ijms21228810 PMC7699902 33233840

[47] Simic, P. & Babitt, J. L. Regulation of FGF23: beyond bone. *Curr. Osteoporos. Rep.* **19**, 563–573. 10.1007/s11914-021-00703-w (2021). 34757587 10.1007/s11914-021-00703-w PMC8958553

[48] Khairallah, P. et al. Acid load and Phosphorus Homeostasis in CKD. *Am. J. Kidney Diseases: Official J. Natl. Kidney Foundation*. **70**, 541–550. 10.1053/j.ajkd.2017.04.022 (2017). 10.1053/j.ajkd.2017.04.022 PMC5804342 28645705

[49] Duong, C. N. et al. Bioavailability of phosphorus and kidney function in the Jackson Heart Study. *Am. J. Clin. Nutr.* **116**, 541–550. 10.1093/ajcn/nqac116 (2022). 35511217 10.1093/ajcn/nqac116 PMC9348986

[50] Calvo, M. S., Dunford, E. K. & Uribarri, J. Industrial Use of phosphate food additives: a mechanism linking Ultra-processed Food Intake to Cardiorenal Disease Risk? *Nutrients*. **15**10.3390/nu15163510 (2023). 10.3390/nu15163510 PMC10459924 37630701

[51] Ferraro, S. et al. Fibroblast growth factor 23: translating analytical improvement into clinical effectiveness for tertiary prevention in chronic kidney disease. *Clin. Chem. Lab. Med.* **60**, 1694–1705. 10.1515/cclm-2022-0635 (2022). 36008874 10.1515/cclm-2022-0635

[52] Inker, L. A. et al. New Creatinine- and cystatin C-Based equations to Estimate GFR without Race. *N. Engl. J. Med.* **385**, 1737–1749. 10.1056/NEJMoa2102953 (2021). 34554658 10.1056/NEJMoa2102953 PMC8822996

