# Correction: Young adults’ circulating FGF23 and α-klotho and their relationship with habitual dietary acid load and phosphorus intake during growth

**DOI:** 10.1038/s41598-025-20022-9

**Published:** 2025-09-16

**Authors:** Luciana Peixoto Franco, Seyedeh-Masomeh Derakhshandeh-Rishehri, Ute Nöthlings, Michaela F. Hartmann, Christian Herder, Hermann Kalhoff, Stefan A. Wudy, Thomas Remer

**Affiliations:** 1 DONALD Study Center, Nutritional Epidemiology, Institute of Nutrition and Food Science, University of Bonn, Heinstück 11, 44225 Dortmund, Germany; 2 Institute of Nutrition and Food Sciences, Nutritional Epidemiology, University of Bonn, Bonn, Germany; 3 Laboratory for Translational Hormone Analytics, Peptide Hormone & Immunoassay Unit, Pediatric Endocrinology & Diabetology, Center of Child and Adolescent Medicine, Justus Liebig University, Giessen, Germany; 4 Institute for Clinical Diabetology, German Diabetes Center (DDZ), Leibniz Center for Diabetes Research at Heinrich Heine University Düsseldorf, Düsseldorf, Germany; 5 German Center for Diabetes Research (DZD), Partner Düsseldorf, München-Neuherberg, Germany; 6 Department of Endocrinology and Diabetology, Medical Faculty, University Hospital Düsseldorf, Heinrich Heine University Düsseldorf, Düsseldorf, Germany; 7 Research Department of Child Nutrition, St. Josef-Hospital, University Hospital of Pediatrics and Adolescent Medicine, Ruhr-University Bochum, Bochum, Germany; 8 Pediatric Clinic Dortmund, Dortmund, Germany

Correction to: *Scientific Reports* 10.1038/s41598-024-79636-0, published online 13 November 2024

The original version of this Article contained an error in the Abstract.

“After adjusting for growth period-related covariates and adulthood confounders only for P-In during growth, i.e., PO4-Ex, but not for NAE and uPRAL, a significant positive association (*p* = 0.03) with FGF23 and an inverse trend (*p* = 0.10) with the FGF23-α-klotho ratio were observed.”

now reads:

“After adjusting for growth period-related covariates and adulthood confounders only for P-In during growth, i.e., PO4-Ex, but not for NAE and uPRAL, a positive association (*p* = 0.03) with FGF23 was observed."

The original Article has been corrected.

