# Supplementary material for: Young adults’ circulating FGF23 and α-klotho and their relationship with habitual dietary acid load and phosphorus intake during growth
Source: Sci Rep. 2024 Nov 13;14:27784. doi: 10.1038/s41598-024-79636-0 (PMC11561314; doi:10.1038/s41598-024-79636-0)
Supplement: Supplementary file 1 — Supplementary Material 1 [file 41598_2024_79636_MOESM1_ESM.pdf]

**Supplementary table S1.** Prospective relationship of renal biomarker of habitual dietary phosphorus intake during growth with the FGF23/klotho ratio in adulthood [a]

| **FGF23/klotho ratio** [b] | | ***β (95% CI)*** | ***$R^2$*** | ***P*** |
|---|---|---|---|---|
| Exposure PO4-SDS | | | | |
| | Model I [c] | 0.16 (-0.04, 0.37) | 0.08 | 0.11 |
| | Model II [d] | 0.20 (-0.01, 0.40) | 0.12 | 0.06 |
| | Model III [e] | 0.17 (-0.03, 0.38) | 0.15 | 0.10 |

Abbreviations: PO4-SDS; individual means of standard deviation scores of 343 children's and adolescents´ 24-h phosphate excretion.

[a] Results obtained from step-wise multi linear regression analyses.

[b] $R^2$ denotes overall explained variability of the model and P denotes the P value for the exposure PO4-SDS of the respective model.

[c] Model I adjusted for sex, adult age, and 24-h urinary urea nitrogen excretion.

[d] Model II adjusted for variables in model I plus additional childhood and adolescent means of SDSs of 24-h urinary excretion variables: osmolality and calcium.

[e] Model III adjusted for variables in model II plus adults' blood parameters: PTH, LDL/HDL ratio, albumin, and phosphate; HOMA-IR and calcium did not met the criteria for model inclusion
