# Supplementary material for: Young adults’ circulating FGF23 and α-klotho and their relationship with habitual dietary acid load and phosphorus intake during growth
Source: Sci Rep. 2024 Nov 13;14:27784. doi: 10.1038/s41598-024-79636-0 (PMC11561314; doi:10.1038/s41598-024-79636-0)
Supplement: Supplementary file 2 — Supplementary Material 2 [file 41598_2024_79636_MOESM2_ESM.pdf]

Supplementary table S2. Sensitivity analysis of the relationship of 24-h urinary phosphate excretion during growth with adult circulating FGF23 in a reduced number of study participants (n=264) all with available measured circulating proinflammatory markers

**Exposure Phosphate (PO4)** [a]		***Q1***	***Q2***	***Q3***	***Q4***	***Q5***	R^2	***P***
PO4-SDS (mmol/d) [b]		-0.70 (-0.76, -0.65)	-0.36 (-0.42, -0.30)	-0.10 (-0.17, -0.04)	0.25 (0.18, 0.32)	0.90 (0.82, 0.99)		
PO4, Median-BSA (mmol/d/1.73 m^2) [c]		23.89 (23.12, 24.67)	25.98 (25.17, 26.78)	27.12 (26.31, 27.93)	28.76 (27.89, 29.63)	33.60 (32.38, 34.83)		
**Outcome**								
FGF23, adjusted mean (pmol/L) [d]	Model I [e]	1.36 (0.92, 1.80)	1.49 (1.19, 1.80)	1.47 (1.23, 1.72)	1.61 (1.34, 1.88)	1.65 (1.13, 2.18)	0.14	0.024
	Model II [f]	1.35 (0.91, 1.78)	1.48 (1.18, 1.78)	1.47 (1.23, 1.72)	1. 57 (1.30, 1.84)	1.73 (1.20, 2.25)	0.18	0.008
	Model III [g]	1.24 (0.80, 1.69)	1.43 (1.13, 1.73)	1.48 (1.24, 1,73)	1.58 (1.31, 1.85)	1.79 (1.26, 2.32)	0.23	0.016
	Model IV [h]	1.23 (0.78, 1.67)	1.42 (1.12, 1.72)	1.49 (1.25, 1.73)	1.55 (1.28, 1.82)	1.79 (1.26, 2.32)	0.25	0.030

Abbreviations: PO4, phosphate; SDS, standard deviation score; BSA, body surface area

[a] R^2 denotes overall explained variability of the model and P denotes the P value for the exposure PO4-SDS of the respective model.

[b] Values are expressed as mean and 95% confidence interval (CI) of the respective quintile.

[c] PO4, median-BSA: values are derived from each individual’s median 24-h PO4 excretion corrected for BSA (median of all collections of the respective individual). Values are expressed as median and inter-quartile range (IQR) of the respective quintile.

[d] Values are expressed as mean and 95% confidence interval (CI).

[e] Model I adjusted for sex, adults’ age, and 24-h urinary urea nitrogen excretion.

[f] Model II adjusted for the variables in model I plus additional childhood & adolescent means of SDSs of nutrition-related 24-h urinary biomarkers (osmolality, calcium, salt, and pH).

[g] Model III adjusted for variables in model II plus adults´ blood-derived-parameters (HOMA-IR, uric acid, PTH, and LDL/HDL ratio); corrected calcium and phosphate blood levels were also considered but did not fulfill the criteria for model inclusion.

[h] Model IV adjusted for variables in model III plus adults’ circulating proinflammatory marker IL-6 (significance level of included IL-6 in model IV: p=0.012).
