# Supplementary material for: Young adults’ circulating FGF23 and α-klotho and their relationship with habitual dietary acid load and phosphorus intake during growth
Source: Sci Rep. 2024 Nov 13;14:27784. doi: 10.1038/s41598-024-79636-0 (PMC11561314; doi:10.1038/s41598-024-79636-0)
Supplement: Supplementary file 3 — Supplementary Material 3 [file 41598_2024_79636_MOESM3_ESM.pdf]

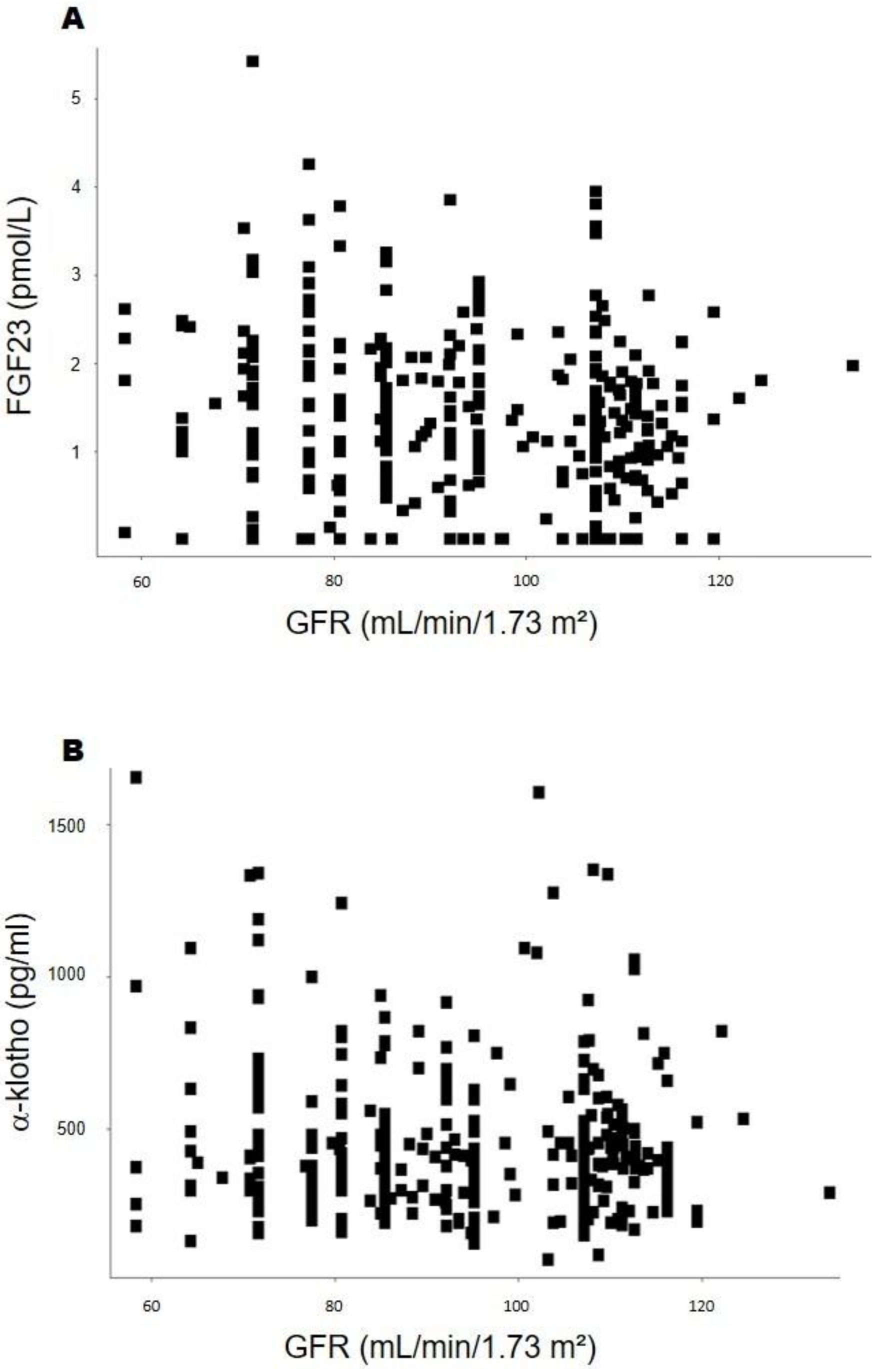

Supplementary Figure S1. Scatterplot of the relationship of estimated glomerular filtration rate (eGFR according to EKFC equations) with FGF23 (**A**) and α-klotho (**B**) in young, healthy adults. According to the Chronic Kidney Disease Epidemiology Collaboration (CKD-EPI) creatinine-based equations, all participants exceeded 60 mL/min/1.73 m².
